# Genetic variants of VEGFR-1 gene promoter in acute myocardial infarction

**DOI:** 10.1186/s40246-019-0243-1

**Published:** 2019-11-19

**Authors:** Haihua Wang, Shufang Zhang, Na Wang, Jie Zhang, Mingkai Chen, Xiaohui He, Yinghua Cui, Shuchao Pang, Bo Yan

**Affiliations:** 1 Shandong Provincial Key Laboratory of Cardiac Disease Diagnosis and Treatment, Affiliated Hospital of Jining Medical University, Jining Medical University, 89 Guhuai Road, Jining, 272029 Shandong China; 2 Division of Cardiology, Affiliated Hospital of Jining Medical University, Jining Medical University, Jining, Shandong China; 3 Division of Laboratory Medicine, Affiliated Hospital of Jining Medical University, Jining Medical University, Jining, Shandong China; 4 Cardiac Care Unit, Affiliated Hospital of Jining Medical Uniiversity, Jining Medical University, Jining, Shandong China

**Keywords:** Acute myocardial infarction, VEGFR-1, DNA sequence variants, Genetics, Promoter

## Abstract

**Background:**

Coronary artery disease (CAD) including acute myocardial infarction (AMI) is a common complex disease caused by atherosclerosis. Vascular epithelial growth factor receptor-1 (VEGFR-1) stimulates angiogenesis and vascular permeability, and functions as a decoy to sequester VEGF and prevent initiation of intracellular signaling. VEGFR-1 knockout mice exhibit significantly higher mortality due to heart failure, cardiac hypertrophy, and cardiac dysfunction. An evident increase in macrophage infiltration and cardiac fibrosis are also observed after transverse aortic constriction. Therefore, VEGFR-1 gene variants may be involved in CAD. In this study, VEGFR-1 gene promoter was genetically and functionally analyzed in large cohorts of AMI patients and ethnic-matched controls.

**Results:**

A total of 16 DNA sequence variants (DSVs) including six single-nucleotide polymorphisms (SNPs) were found in the VEGFR-1 gene promoter and 5′-untranslated region. Five novel DSVs and one SNP were only identified in AMI patients group. These DSVs and SNP significantly altered the transcriptional activity of the VEGFR-1 gene promoter in both HEK-293 and H9c2 cells (*P* < 0.05). Further electrophoretic mobility shift assay indicated that the DSVs and SNPs evidently affected the binding of transcription factors.

**Conclusions:**

The genetic variants in VEGFR-1 gene identified in AMI patients may alter the transcriptional activity of the VEGFR-1 gene promoter and change VEGFR-1 level, contributing to AMI development.

## Background

Coronary artery disease (CAD) including acute myocardial infarction (AMI) is a common complex disease, which is mainly caused by atherosclerosis, an inflammatory and metabolic disease. The known risk factors for CAD include hypertension, obesity, aging, smoking, hyperlipidemia, diabetes, inflammation, and family history. A great number of common genetic variants for CAD have been identified, which only contribute to ~ 10% of heritability [1–6]. Recently, genetic variants of low and rare frequencies have been proposed to contribute to CAD and AMI.

Proper development of the coronary vascular system is critical to deliver blood supply for myocardial growth and to ensure efficient cardiac contraction [7]. Vascular endothelial growth factor receptor-1 (VEGFR-1) is a transmembrane protein, including two isoforms, a full length transmembrane VEGFR-1/Flt-1 and soluble VEGFR-1 (sVEGFR-1/sFlt-1), which lacks the transmembrane and cytoplasmic kinase domains [8]. VEGFR-1 binds to vascular endothelial growth factor A (VEGF-A) and placental growth factor (PLGF) to stimulate angiogenesis and vascular permeability [9]. VEGFR-1 gene is homogeneously expressed in vascular epithelial cells of neonatal mouse hearts, and in coronary vessels of adult hearts [10]. sFlt-1 levels are dramatically stimulated in most cases of hypoxia [11].

Mounting evidence indicates that the function of VEGF family members extends beyond blood vessel formation. VEGFR-1 activation by VEGF-B prevents loss of cardiac mass and promotes maintenance of cardiac contractility over time [12]. The VEGFR-1 gene knockout mice have significantly higher mortality due to heart failure, and showed evident cardiac hypertrophy and cardiac dysfunction, which was accompanied by a significant increase in macrophage infiltration and cardiac fibrosis after transverse aortic constriction [13]. sVEGFR-1 regulates the availability of free VEGF and PLGF in peripheral circulation [14].

Recently, sVEGFR-1 has been implicated in the pathogenesis of cardiovascular disease [15–22]. Acting as a negative regulator of angiogenesis, sVEGFR-1 binds to VEGF, inhibiting the angiogenic action of VEGF in arterial endothelium [23]. Elevated levels of sVEGFR-1 have been suggested as a predictor for mortality in AMI patients [24]. Therefore, we speculated that altered expression of VEGFR-1 gene, caused by the DNA sequence variants (DSVs) with its regulatory regions, may contribute to the CAD development. In this study, we genetically analyzed the VEGFR-1 gene promoter in large cohorts of AMI patients and healthy controls.

## Results

### Genetic variants identified in the VEGFR-1 gene promoter and 5′-UTR

**Fig. 1:**
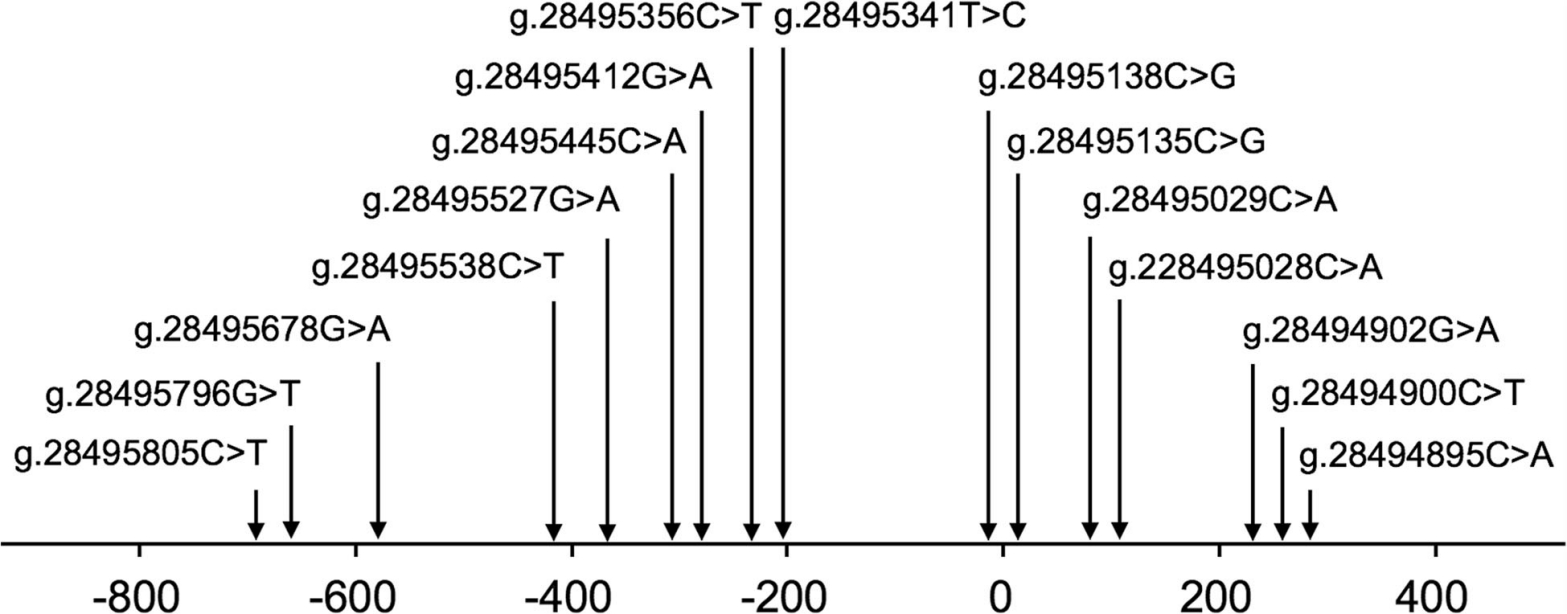
Locations of the identified DSVs and SNPs within the VEGFR-1 gene promoter and 5′-UTR. The genetic variants are named according to the genomic DNA sequence of the human VEGFR-1 gene (Genbank accession number NC_000013.11). The transcription start site is at the position of 28495128 in the first exon

**Fig. 2:**
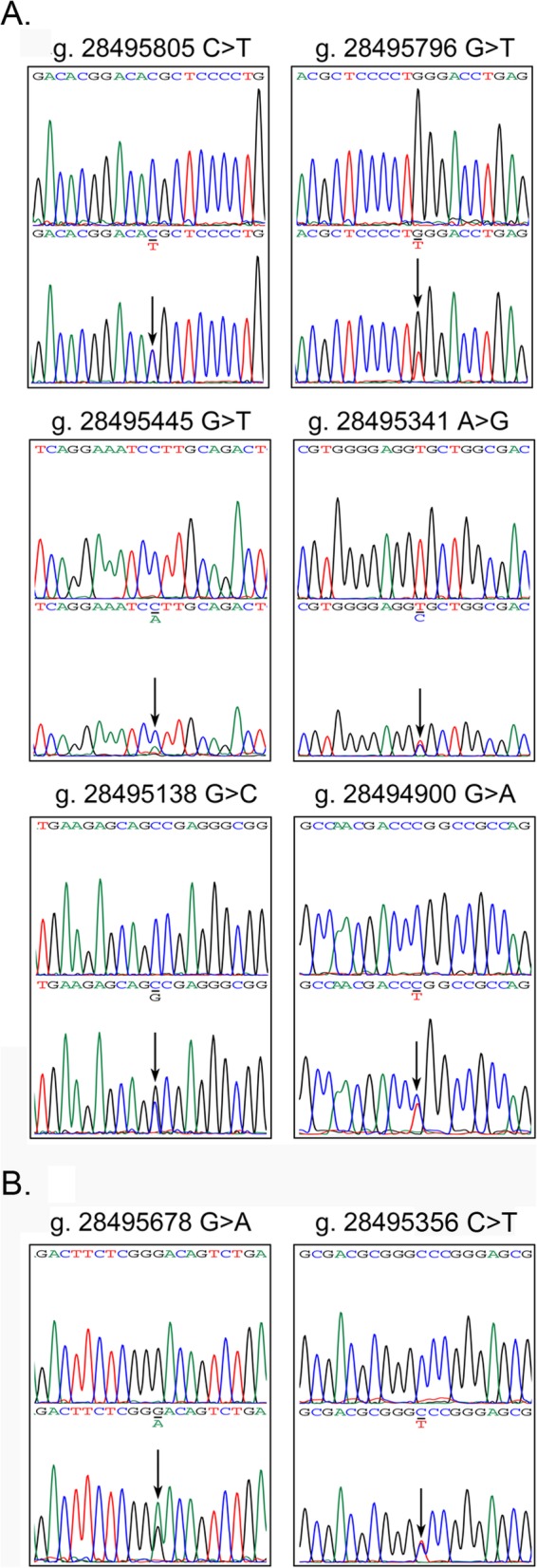
Sequencing chromatograms of the DSVs and SNPs. **a** Sequencing chromatograms of the DSVs and SNPs only identified in AMI patients, g.28495805C>T (rs111458691), g.28495796 G>T, g.28495445 G>T, g.28495341 A>G, g.28495138 G>C, and g.28494900 G>A. **b** Sequencing chromatograms of the DSVs identified only in controls, g.28495678 G>A and g.28495356G>A. Top panels show wild-type and bottom panels heterozygous sequences, which are marked with arrows

In this study population, a total of 16 DSVs were identified in the VEGFR-1gene promoter and 5′-untranlated region (5′-UTR), including ten novel heterozygous DSVs and six single-nucleotide polymorphisms (SNPs). Distributions and locations of the genetic variants are summarized in Table 1 and Fig. 1. Five novel heterozygous DSVs, g.28495796G > T, g.28495445G > T, g.28495341A > G, g.28495138G > C and g.28494900G>A, and one SNP, g.28495805C>T (rs111458691), were identified in six AMI patients but in none of the controls. The DNA sequencing chromatograms of these novel DSVs were shown in Fig. 2a. Two novel heterozygous DSVs, g.28495678G>A and g. 28495356G>A, were only found in two healthy controls. The DNA sequencing chromatograms of these novel DSVs were shown in Fig. 2b. The other three novel DSVs (g.28495029G>T, g.28495028C>A, and g. 28494895G>T) and five SNPs [g.28495538C>T (rs17086747), g. 28495527G>A (rs117031451), g.28495412C>T (rs36204411), g.28495135G>C (rs17086745), and g.28494902C>T (rs55927955)] were found in both AMI patients and controls with similar frequencies (*P* > 0.05). In addition, distribution of all SNPs was in Hardy-Weinberg equilibrium in both groups of AMI patients and healthy controls (*P* > 0.05).

**Table 1:** Genetic variants in the VEGFR-1 gene promoter identified in AMI patients and controls

DSVs and SNPs	Genotype	Location^a^	Controls (*n* = 414)	AMI (*n* = 386)	*P* value
g.28495805C>T (rs111458691)	CT	− 677 bp	0	1	–
g.28495796G>T	GT	− 668 bp	0	1	–
g.28495678G>A	GA	− 550 bp	1	0	–
g.28495538C>T (rs17086747)	CC	− 410 bp	388	357	0.574
	CT		26	28	
	TT		0	1	
g.28495527G>A (rs117031451)	GG	− 399 bp	380	360	0.748
	GA		33	25	
	AA		1	1	
g.28495445G>T	GT	− 317 bp	0	1	–
g.28495412C>T (rs36204411)	CC	− 284 bp	388	357	0.574
	CT		26	28	
	TT		0	1	
g.28495356G>A	GA	− 228 bp	1	0	–
g.28495341A>G	AG	− 213 bp	0	1	–
g.28495138G>C	GC	− 10 bp	0	1	–
g.28495135 G>C (rs17086745)	GG	− 7 bp	388	357	0.574
	GC		26	28	
	CC		0	1	
g.28495029G>T	GT	+ 99 bp	1	1	1.000
g.28495028C>A	CA	+ 100 bp	1	1	1.000
g.28494902C>T (rs55927955)	CC	+ 226 bp	355	343	0.162
	CT		58	43	
	TT		1	0	
g.28494900G>A	GA	+ 228 bp	0	1	–
g.28494895G>T	GT	+ 233 bp	1	1	1.000

^a^DSVs are located upstream (−) and downstream (+) to the transcription start site at 28495128 of VEGFR-1 gene (NC_000013.11). *AMI* acute myocardial infarction, *DSV* DNA sequence variant, − not applicable

### Putative binding sites for transcription factors affected by genetic variants

To determine whether the genetic variants affect putative binding sites for transcription factors, the VEGFR-1 gene promoter was analyzed with JASPAR program (http://jaspar.genereg.net/) [25].

The results showed that genetic variants found only in AMI patients or healthy controls may abolish or create binding sites for transcription factors. The analysis data were summarized in Table 2. Among these transcription factors, a Forkhead box protein O3 (FOXO3) gene SNP (rs2802292) has been reported as a protective factor against CAD mortality [26].

**Table 2:** Predicted binding sites for transcription factors and promoter activity affected by DSVs and SNPs

DSVs/SNPs	Binding cites for transcription factors		Promoter activity	AMI/CTR
	Creating	Abolishing		
g.28495805C>T (rs111458691)	ELF5, NFE2L1:MafG	EGR2	↑	AMI
g.28495796G>T	–	ESRRA, NR2C2, PAX5, PPARG::RXRA, ZEB1	No change	AMI
g.28495678G>A	NFATC1	TFE3	No change	CTR
g.28495445G>T	FOXO3, NFE2L1::MafG	NFKB1	↓	AMI
g.28495356G>A	HINFP	E2F4, TFAP2C	No change	CTR
g.28495341A>G	EGR1, NRF1	BRCA1, HIF1A::ARNT	No change	AMI
g.28495138G>C	–	NHLH1, HIF1A::ARNT	↓	AMI
g.28494900G>A	NF1C	–	↑	AMI

### Functional analysis of the DSVs by dual-luciferase reporter assay

The expression vectors for luciferase reporter gene were constructed with wild-type and variant VEGFR-1 gene promoters. The expression vector for wild-type VEGFR-1 gene promoter was designated as pGL3-WT. The variant expression vectors for the DSVS in AMI patients included pGL3-28495805 T, pGL3-28495796 T, pGL3-28495445 T, pGL3-28495341G, pGL3-28495138C, and pGL3-28494900A. The variant expression vectors for the DSVs in healthy controls included pGL3-28495356A and pGL3-28495678A. Empty pGL3-basic vector was used as a negative control. Transfected human embryonic-kidney cell line (HEK-293) and rat cardiomyocyte line (H9c2) cells were collected and dual-luciferase activities were assayed. Transcriptional activity of the wild-type VEGFR-1 gene promoter was set as 100%. The relative transcriptional activity of the variant VEGFR-1gene promoter was calculated compared to that of wild type VEGFR-1 gene promoter.

**Fig. 3:**
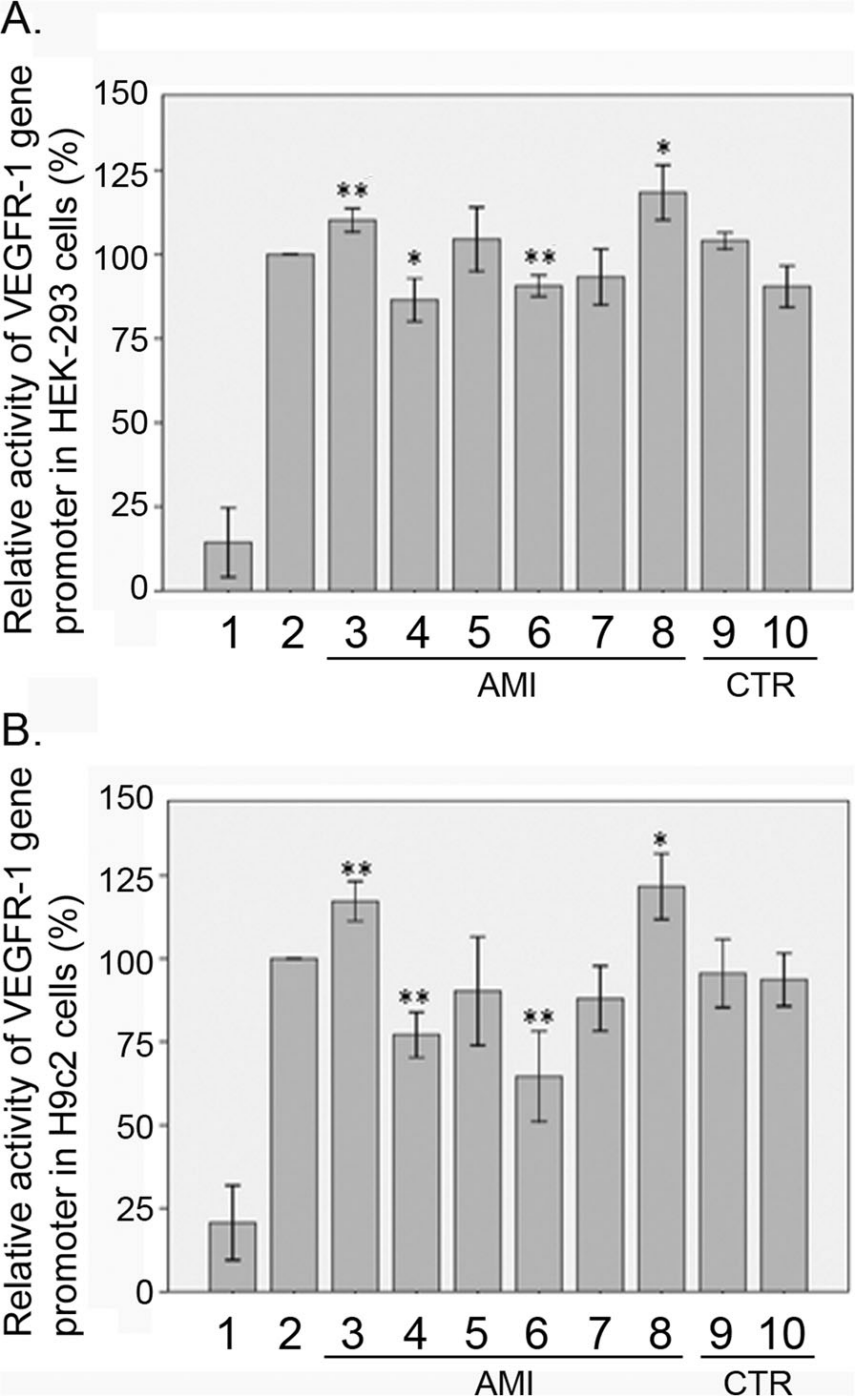
Relative transcriptional activity of wild type and variant VEGFR-1 gene promoters. Wild-type and variant VEGFR-1 gene promoters were cloned into reporter gene vector pGL3 and transfected into cultured cells. The transfected cells were collected, and dual-luciferase activities were assayed. Empty vector pGL3-basic was used as a negative control. Transcriptional activity of the wild-type VEGFR-1 gene promoter was designed as 100%. Relative activities of variant VEGFR-1 gene promoters were calculated. **a** Relative activities of wild type and variant VEGFR-1 gene promoters in HEK-293 cells. **b** Relative activities of wild-type and variant VEGFR-1 gene promoters in H9c2 cells. Lanes 1, pGL3-basic; 2, pGL3-WT; 3, pGL3-28494900A; 4, pGL3-28495138C; 5, pGL3-28495341G; 6, pGL3-28,495,445 T; 7, pGL3-28,495,796 T; 8, pGL3-28,495,805 T; 9, pGL3-28495356A; and 10, pGL3-28495678A. *WT* wild type, *AMI* AMI patients, *CTR* healthy controls. *P < 0.05, compared to pGL3-WT; **P < 0.01, compared to PGL3-WT

In HEK-293 cells, the DSVs (g.28495445G>T, g.28495138G>C) that were identified only in AMI patients significantly decreased activity of the VEGFR-1gene promoter (*P* = 0.004 and *P* = 0.032, respectively). In contrast, the SNP [g.28495805C>T (rs111458691)] and the DSV (g.28494900G>A) identified in AMI patients significantly increased the transcriptional activity of the VEGFR-1 gene promoter (*P* = 0.021 and *P* = 0.004, respectively). In contrast, the DSVs (g.28495341A>G and g.28495796G>T) identified in AMI patients did not affect the transcriptional activity of the VEGFR-1 gene promoter (*P* > 0.05). As expected, the DSVs found only in controls (g. 28495356G>A and g.28495678G>A) did not significantly alter activity of the VEGFR-1 gene promoter (*P* > 0.05) (Fig. 3a).

To further investigate the tissue-specificity, the transcriptional activities of the variant VEGFR-1 gene promoters were examined in H9c2 cells. The DSVs (g. 28495445G>T and g. 28495138G>C) significantly decreased activity of the VEGFR-1 gene promoter (*P* = 0.009 and *P* = 0.002, respectively). The SNP [g.28495805C>T (rs111458691)] and the DSV (g. 28494900G>A) significantly increased the transcriptional activity of the VEGFR-1 gene promoter (*P* = 0.026 and *P* = 0.006 respectively). Similarly, the DSVs (g. 28495356G>A and g. 28495678G>A) found only in controls did not significantly alter activity of the VEGFR-1 gene promoter (*P* > 0.05) (Fig. 3b). The effects of DSVs and SNPs on VEGFR-1 gene promoter transcriptional activity were also shown in Table 2. Taken together, the DSVs and SNPs in the VEGFR-1 gene promoter identified in AMI patients affected the activity of the VEGFR-1 gene promoter in both HEK-293 cells and H9c2 cells, which were non-tissue specific.

### The binding sites for transcription factors affected by the DSVs

**Fig. 4:**
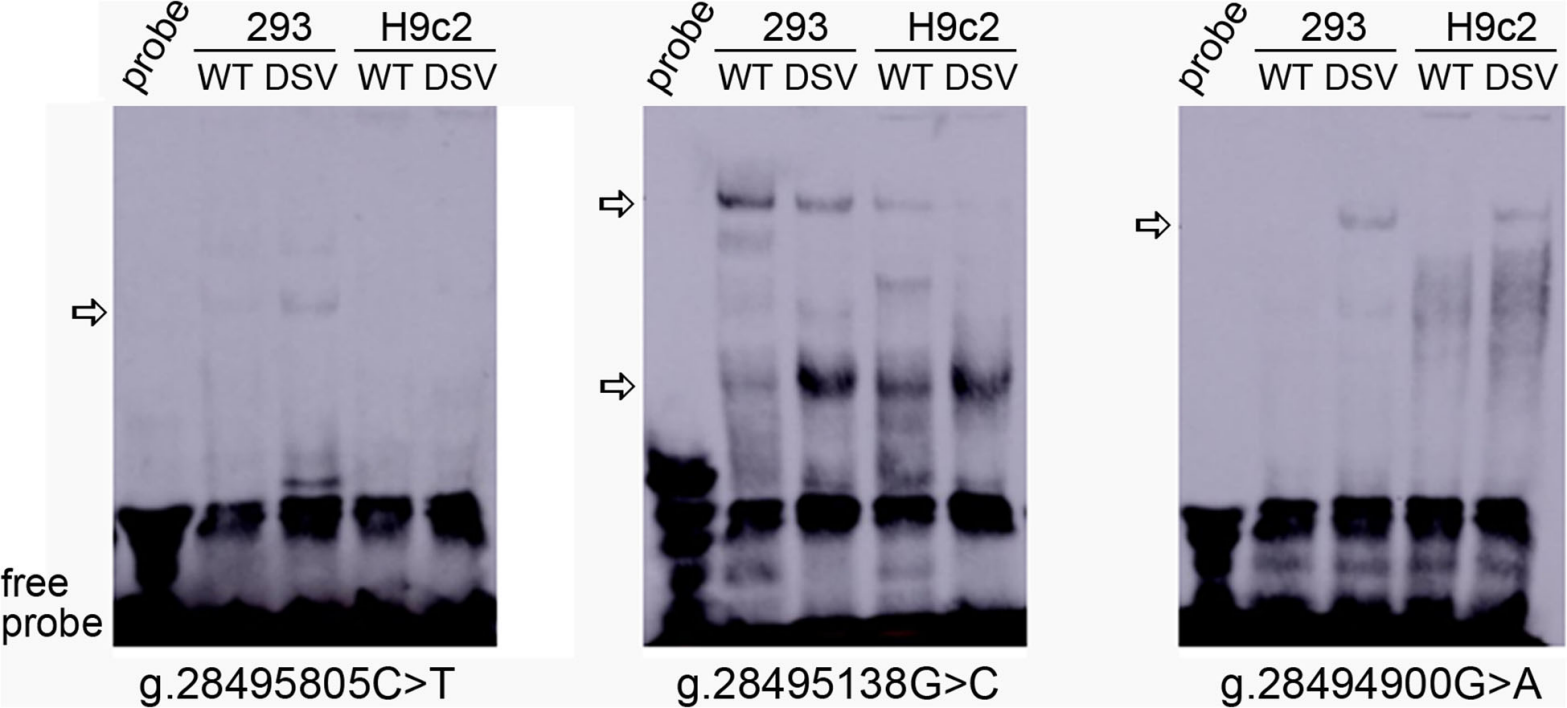
EMSA of biotin-labeled oligonucleotides. Wild-type and variant oligonucleotides (30 bp) were designed and labeled with biotin for genetic variants identified in AMI patients, including g.28495805C>T(rs111458691), g.28495138G>C, and g.28494900G>A. EMSA was conducted with biotinylated oligonucleotides and the nuclear extracts from HEK-293 and H9c2 cells. Free probe was marked with an arrow at the bottom. The affected binding for transcription factors was marked with an open arrow

To explore whether the genetic variants affected the binding sites for transcription factors, EMSA was performed with wild-type or variant oligonucleotides (Table 3). As shown in Fig. 4, the SNP g.28495805C>T (rs111458691) weakly enhanced the binding for a transcription factor in 293 cells, which was not observed in H9c2 cells. The DSV g.28495138 G>C significantly weakened the binding of a transcription factor and enhanced the binding of another transcription factor in both 293 and H9c2 cells. The DSV g.28494900G>A created a binding of a transcription factor in both 293 and H9c2 cells. However, the DSVs g.28495796G>T, g.28495445 G>T, and g.28495341A>G did not influence the binding of transcription factors, the results of which were not shown. Therefore, these genetic variants may alter the transcription of VEGFR-1 gene through affecting the binding of transcription factors.

**Table 3:** Double-stranded biotinylated oligonucleotides containing DSVs in AMI for EMSA

DSVs	Oligonucleotide sequences
g.28495805C>T (rs111458691)	5′-AAAAAGACACGGACA(C/T)GCTCCCCTGGGACC-3′
g.28495796G>T	5′-CGGACACGCTCCCCT(G/T)GGACCTGAGCTGGT-3′
g.28495445G>T	5′-GGAGGGAGTCTGCAA(G/T)GATTTCCTGAGCGC-3′
g.28495341A>G	5′-GCCCGCGTCGCCAGC(A/G)CCTCCCCACGCGCG-3′
g.28495138G>C	5′-TCGCCCCCGCCCTCG(G/C)CTGCTCTTCATCGA-3′
g.28494900G>A	5′-CGGACTCTGGCGGCC(G/A)GGTCGTTGGCCGCG-3′

## Discussion

To date, the VEGFR-1 gene has been implicated in human diseases. Changes in VEGFR-1 expression have been reported in human melanoma cells to stimulate cell proliferation, chemotaxis, and extracellular matrix invasion [27]. A recent study reveals that VEGFR-1 recruits CD133^+^ progenitors to inhibit local inflammation [28]. A decrease in VEGFR-1 has been associated with osteoarthritis pain [29]. sVEGFR-1 levels are increased in preeclampsia—a pathological condition of pregnancy [30]. Previous studies have shown that elevated sFlt-1 contributes to the development of heart failure. sFlt-1 levels in plasm are directly correlated with the severity of heart failure, and also strongly associated with poor outcomes in patients with heart failure [15, 19]. In this study, we found five novel heterozygous DSVs and one SNP in the VEGFR-1 gene promoter and 5′-UTR in six AMI patients. These DSVs and SNP significantly affected the transcriptional activity of the VEGFR-1 gene promoter by influencing the binding of transcription factors. Therefore, the genetic variants in VEGFR-1 gene may alter transcriptional activity of VEGFR-1 gene promoter and change VEGFR-1 level, contributing to AMI development as a risk factor.

Human VEGFR-1 gene has been localized to chromosome 13p12 and encodes two isoforms: a full length transmembrane VEGFR-1/Flt-1, and soluble VEGFR-1 (sVEGFR-1/sFlt-1). While expression of transmembrane VEGFR-1 and VEGF-A is increased by hypoxia, it is not clear whether sVEGFR-1 expression is downregulated or upregulated by reduced oxygen concentration [31, 32]. Ado downregulates sVEGFR-1 and upregulates VEGFR-1 production to stimulate angiogenesis in the heart [33]. Mouse studies have shown that after myocardial infarction, expression of VEGFR-1 is induced in the regenerating coronary vessels at day 7 and is maintained in the newly formed coronary vessels until 30 days after myocardial infarction, recapitulating their expression kinetics during development [10].

Numerous studies have demonstrated that stimulation of angiogenesis is beneficial to ischemic and infarcted heart [34–37]. Collateral growth during ischemia requires proliferation of endothelial cells, recruitment of leukocytes and endothelial progenitor cells, and remodeling of the extracellular matrix. In the process, VEGFs play a key role via binding to VEGFR-1 and VEGFR-2 selectively [38–43]. VEGFR-1 has a primary role in embryonic angiogenesis. Mice carrying a homozygous deletion of VEGFR-1 intracellular kinase domain showed correct development of blood vessels [44]. While VEGFR-1 negatively regulates endothelial cell differentiation during development, it promotes angiogenesis under certain conditions in adult tissues, especially in ischemic tissues. Transmembrane VEGFR-1 and its selective ligands PLGF and VEGF-B have been highly involved in pathological angiogenesis [45, 46]. Under certain circumstances upon stimulation with PLGF, a VEGFR-1-specific ligand, VEGFR-1 may heterodimerize with VEGFR-2 leading to transactivation of VEGFR-2 and angiogenesis [47]. Overexpression of PLGF in transgenic mice is also reported to promote angiogenesis [48]. These findings suggest that when VEGFR-1 is paired with VEGFR-2 via a mechanism of heterodimerization, its activity positively regulates angiogenesis. Therefore, changed VEGFR-1 level may interfere with angiogenesis via above pathways.

## Conclusions

In this study, we found several functional genetic variants in VEGFR-1 gene in AMI patients. These genetic variants may change VEGFR-1 level, contributing to AMI development via affecting angiogenesis. These data warrant for further investigation of the molecular mechanisms by which the genetic variants change VEGFR-1 gene expression.

## Methods

### AMI patients and healthy controls

All AMI patients (*n* = 386; age24–86 years, mean age 62.24 years; males 221 and females 165) were recruited from the Cardiac Care Unit in Division of Cardiology, Affiliated Hospital of Jining Medical University, Jining Medical University, Jining, Shandong, China. AMI patients were diagnosed according to clinical manifestations, abnormal electrocardiogram, and elevated levels of plasma cardiac necrosis makers. Ethnic-matched healthy controls (*n* = 414; age 20–84 years, mean age 51.33 years; males 235 and females 179) were from Physical Examination Center in the same hospital. Healthy controls with familial CAD histories were excluded from the study. This study was approved by the Human Ethics Committee of the Affiliated Hospital of Jining Medical University. Informed consents were obtained from all participates.

### Direct DNA sequencing

Peripheral leukocytes were isolated venous blood (5.0 ml). Genomic DNAs in leukocytes were extracted with DNeasy Blood and Tissue Kit (QIAGEN, Valencia, CA, USA). The putative VEGFR-1 gene promoter and 5′-UTR (− 1022 bp ~ + 284 bp to the transcription start site) was analyzed. Two DNA fragments, 770 bp (− 1092 bp ~ − 338 bp) and 788 bp (− 479 bp ~ + 308 bp), which overlappingly covered the VEGFR-1 gene promoter and 5′-UTR, were generated by polymerase chain reaction (PCR) with Taq DNA polymerase PCR master mix (Promega Corporation, Madison, WI, USA). Genomic DNAs (100 ng) were used as PCR template. PCR primers were designed with the genomic sequence of human VEGFR-1 gene (GenBank accession number, NC_000013.11) (Table 4). PCR products were bi-directionally sequenced by Sangon Biotech Co., Ltd. (Shanghai, China) with 3730 DNA Analyzer (Applied Biosystems, Foster City, CA, USA). DNA sequences were then compared with the wild-type VEGFR-1 gene promoter and 5′-UTR using DNAMAN program (version 5.2.2; Lynnon BioSoft, Quebec, Canada), and DNA variants were identified. The DNA variants in the VEGFR-1 gene promoter were first analyzed using JASPAR program to predict their effects on binding sites for transcription factors, which were further experimentally confirmed.

**Table 4:** PCR primers for the VEGFR-1 gene promoter and 5′-UTR region

PCR primers	Sequences	Location	Position	Products
Sequencing				
VEGFR-1-F1	5′-ACATCCCTCTGACGGGTTCCA-3′	28496221	− 1092 bp	770 bp
VEGFR-1-R1	5′-GTAAGCCGGGTGGAGGGAGT-3′	28495471	− 338 bp	
VEGFR-1-F2	5′-GCCTCAGTCCTCCGTGCCAAGA-3′	28495608	− 479 bp	788 bp
VEGFR-1-R2	5′-ATGGTCAGCTACTGGGACACCGG-3′	28494821	+ 308 bp	
Functioning				
VEGFR-1-F	5′-(KpnI)-GCTCCGTGCAGCCAGGACGA-3′	28,496,151	− 1022 bp	1307 bp
VEGFR-1-R	5′-(HindIII)-GTGAGCGCGACGCGGCCT-3′	28,494,845	+ 284 bp	

PCR primers are designed based on the genomic DNA sequence of the VEGFR-1 gene (NC_000013.11). The transcription start is at the position of 28495128 (+ 1). *PCR* polymerase chain reaction

### Functional analysis with dual-luciferase reporter assay

Wild-type and variant VEGFR-1 gene promoter and 5′-UTR (1307 bp, − 1022 bp ~ + 284 bp) was generated by PCR and inserted into the KpnI and HindIII sites of a luciferase reporter plasmid (pGL3-basic) to generate expression vectors. PCR primers with KpnI or HindIII sites were shown in Table 4. All expression vectors were confirmed by direct DNA sequencing. Designated expression vectors were transiently transfected into human embryonic kidney cell line [HEK-293, CRL-1573; American Type Culture Collection (ATCC), Manassas, VA, USA] and rat cardiomyocyte cell line (H9c2, CRL-1446; ATCC). HEK-293 and H9c2 cells were cultured at 37 °C in 5% CO_2_ humidified environment. On the day prior to transfection, the cells were seeded in 6-well plates at 40–50% confluence. Designated expression vectors (1.0 μg) and Lipofectamine® (3.0 μl; Thermo Fisher Scientific, Rockford, IL, USA) in 500 ml serum-free medium were used for transfection in each well. The vector expressing Renilla luciferase (pRL-TK; 25 ng, Promega Corporation) was used as an internal control for transfection efficiency. Forty-eight hours after transfection, the transfected cells were collected and luciferases activities were measured using dual-luciferase reporter assay system on a Promega Glomax 20/20 luminometer. Plasmid pGL3-basic was used as a negative control. VEGFR-1 gene promoter activity was expressed as the ratio of luciferase activity over Renilla luciferase activity. Transcriptional activity of wild-type VEGFR-1 gene promoter was set as 100%. All the experiments were repeated at least three times independently, in triplicate.

Nuclear extracts preparation and electrophoretic mobility shift assay (EMSA).

Nuclear extracts were prepared from HEK-293 and H9c2 cells using NE-PER® Nuclear and Cytoplasmic Extraction Reagents (Thermo Fisher Scientific). Protein concentrations of nuclear extracts were determined with Bio-Rad protein assay reagent. EMSA was performed using the LightShift® Chemiluminescent EMSA kit (Thermo Fisher Scientific) following manufacturer’s protocol. Biotinylated double-stranded oligonucleotides were designed on the wild-type and variant DNA sequence. DNA-protein-binding reaction was incubated for 20 min at room temperature with equal amounts of biotinylated oligonucleotides (0.2 pM) and nuclear extracts (3.0 μg). The reaction mixtures were separated on a native 6% polyacrylamide gel at 100 V for 1 h at room temperature, and subsequently transferred onto a nylon membrane at 100 V for 30 min. The oligonucleotides were cross-linked to the membrane using the UV Stratalinker 1800 (Stratagene, Santa Clara, CA, USA) and then detected by chemiluminescence using the LightShift® Chemiluminescent EMSA kit (Thermo Fisher Scientific).

### Statistical analysis

Quantitative data were represented as mean ± SEM, and analyzed using two-way analysis of variance followed by Dunnett’s test. DSV frequencies were analyzed with χ2 test using SPSS v13.0 (SPSS, Inc., Chicago, IL, USA). *P* < 0.05 was considered statistically significant.

## Data Availability

All data generated or analyzed during this study are included in this published article.

## Abbreviations

5′-UTR: 5′-Untranslated region
AMI: Acute myocardial infarction
ARNT: Aryl hydrocarbon receptor nuclear translocator
BRCA1: Breast cancer 1
CAD: Coronary artery disease
CTR: Controls
DSVs: DNA sequence variants E2F4 E2F transcription factor 4
EGR1: Early growth response protein 1
EGR2: Early growth response protein 2
ELF5: E74-like factor 5
ESRRA: Estrogen-related receptor alpha
FOXO3: Forkhead box protein O3
H9c2: Rat cardiomyocyte line
HEK-293: Human embryonic-kidney cell line
HIF1A::ARNT: Heterodimers of HIF-1A (hypoxia-inducible factor 1A) and ARNT (aryl hydrocarbon receptor nuclear translocator)
HINFP: Histone H4 transcription factor
NFATC1: Nuclear factor of activated T-cells 1
NFE2L1: Nuclear factor erythroid-2-like 1
NFE2L1:MafG: Heterodimers of NFE2L1 (nuclear factor erythroid-2-like 1) and a small Maf protein
NFIC: Nuclear factor I-C
NFKB1: Nuclear factor NF-kappa-B 1
NHLH1: Nescient helix-loop-helix 1
NR2C2: Nuclear receptor subfamily 2 group C member 2
NRF1: Nuclear factor E2-related factor 1
PAX5: Paired box transcription factor 5
PLGF: Placental growth factor
PPARG::RXRA: Heterodimers of PPARG (peroxisome proliferator-activated receptor gamma) and RXRA (retinoid X receptor-α)
SNPs: Single-nucleotide polymorphisms
TFAP2C: Transcription factor AP-2 gamma
TFE3: Transcription factor E3
VEGF: Vascular endothelial growth factor
VEGF-A: Vascular endothelial growth factor A
VEGF-B: Vascular endothelial growth factor B
VEGFR-1: Vascular epithelial growth factor receptor-1
VEGFR-1/Flt-1: A full length transmembrane VEGFR-1; sVEGFR-1/sFlt-1, soluble
ZEB1: Zinc finger E-box binding homeobox 1
